# Distal chevron osteotomies enhance patient-reported outcomes for all severity grades of hallux valgus: a cohort study

**DOI:** 10.2340/17453674.2025.44750

**Published:** 2025-10-16

**Authors:** Cyrus D BRODÉN, Ann-Charlott SÖDERPALM, Eva TENGMAN, Nils P HAILER, Maria C CÖSTER

**Affiliations:** 1 Department of Surgical Sciences, Section of Orthopaedics and Hand Surgery, Uppsala University, Uppsala; 2 Department of Orthopaedics and Clinical Sciences, Sahlgrenska Academy, University of Gothenburg, Gothenburg; 3 Department of Community Medicine and Rehabilitation, Umeå University, Umeå, Sweden

## Abstract

**Background and purpose:**

There is limited data on the functional outcome after hallux valgus (HV) surgery. Our study aims to assess 1-year postoperative patient-reported outcomes (PROMs) after a chevron osteotomy (CO) for 3 severity levels, the number of additional surgical interventions during the index procedure, and the association between the presence or absence of internal fixation and PROMs.

**Methods:**

This is a prospective cohort from the Swedish register for foot and ankle surgery (Swefoot), including patients treated with primary CO between 2014 and 2021. HV deformities were classified into 3 severity grades. Preoperative demographic data, additional surgical procedures, and PROMs (Self-reported Foot and Ankle Score [SEFAS] and the EuroQol 5-dimensional 3-level scale [EQ-5D-3L]) were collected both pre- and 1 year post-surgery.

**Results:**

The study included 2,259 HV feet (2,123 patients, mean age 55 (range 15–90) years, 83% women) The mean SEFAS score increased by 11 (95% confidence interval [CI] 9.8–11.8) points from the pre-surgery assessment to the 1-year post-surgery follow-up for the mild HV group, by 9 (CI 9.0–10.0) for the moderate, and by 9 (CI 7.5–9.8) for the severe group. EQ-5D-3L also improved in all 3 groups. For all 3 HV grades, patients treated with fixation demonstrated no statistically significant improvements in SEFAS scores compared with those without fixation.

**Conclusion:**

Distal chevron osteotomy improved 1-year patient-reported outcomes across all grades of hallux valgus. Improvements were observed both with and without internal fixation. In more severe cases, additional procedures such as Akin osteotomy and distal soft tissue release were more commonly performed.

Hallux valgus (HV) may cause considerable pain, footwear problems, functional limitations and reduced health-related quality of life (HRQOL). When non-surgical treatments are insufficient, various surgical procedures are available [1-3]. Distal osteotomies of the first metatarsal are commonly used for mild deformities, while shaft and proximal osteotomies are preferred for more severe cases [4,5]. However, the optimal surgical approach for different grades of deformity remains uncertain.

One specific type of distal osteotomy is the distal V-shaped chevron osteotomy (CO) [6]. The technique involves a V-shaped osteotomy at the distal aspect of the first metatarsal, allowing realignment of the first metatarsal to restore proper alignment of the hallux. The osteotomy is typically fixed using screws to maintain the corrected position during healing [6]. Existing studies on CO are largely retrospective, based on lower-level evidence (Level IV), and involve small sample sizes, raising concerns about their generalizability [5,6]. In addition, it is still unclear whether the osteotomies should be internally fixated.

The primary aim of our study was to evaluate the 1-year postoperative patient-reported outcomes after distal CO in HV cases of varying severity. Secondarily, we investigated whether patient-reported outcomes differ between internally fixated and non-fixated COs and if more severe HV cases are associated with a higher likelihood of additional procedures.

## Methods

### Study design

This observational prospective cohort study was reported in accordance with the Strengthening the Reporting of Observational Studies in Epidemiology (STROBE) guidelines.

### Source of data

We used data from Swefoot, a Swedish national quality register initiated in 2014, which covers 85% of all healthcare facilities in Sweden performing foot and ankle surgeries and has 60% completeness. It collects data on 20 different foot or ankle diagnoses, including HV [7]. The register includes baseline preoperative information such as sex, age, and comorbidities. At the time of the surgical procedure, the surgeon reports the diagnosis, radiographic findings, disease severity, type of surgical procedure, fixation method, and postoperative regimen. Patients are also asked to complete 2 patient-reported outcome measures (PROMs): the Self-reported Foot and Ankle Score (SEFAS) and the EuroQol 5-dimensional 3-level version (EQ-5D 3L) before surgery, and 1 year after surgery. Alongside these PROMs, patients are asked questions concerning aspects like appearance, footwear, strength, and forefoot discomfort. At the 1-year follow up, they also respond to questions about their satisfaction with the procedure’s outcomes and any occurrences of adverse events. For SEFAS, the completeness of PROMs was 76% at the 1-year follow-up.

### Patients

The severity of HV in the register adhered to the classification system by Mann and Coughlin where grade 1 signifies mild disease (intermetatarsal angle [IMA] < 11°, hallux valgus angle [HVA] < 20°), grade 2 moderate disease (IMA 11–16°, HVA 20–40°) and grade 3 denotes severe disease (IMA > 16°, HVA > 40°) [4]. Distal COs were either fixated using a K-wire or screw, with the K-wire removed after 6 weeks, or performed without fixation, meaning no osteosynthesis material was used.

### Patient-reported outcome measures (PROMs)

The SEFAS is a foot- and ankle-specific PROM that encompasses constructs such as pain, functional limitations, and HRQOL. It has been evaluated for both forefoot and hindfoot conditions and is recommended for evaluating HV [8,9]. The summary score ranges from 0 (indicating severe disability) to 48 (reflecting normal function) and is calculated based on responses from 12 questions. The minimal important change (MIC) value, which represents the smallest change in score that patients perceive as important for forefoot disorders, is 5 points [9].

EQ-5D 3L assesses the dimensions mobility, self-care, usual activities, pain/discomfort, and anxiety/depression. Each dimension has 3 levels: no problems, some problems, and extreme problems [10,11]. A summary index is calculated after adjusting for cultural differences. In the present study, the UK EQ-5D tariff is used to compute an index score, with a maximum value of 1.0, which denotes full health.

The response rates for all PROMs in the register are 69% preoperatively and 59% at the 1-year follow-up.

### Statistics

Continuous data was summarized using means and standard deviations (SD). Categorical data is shown in the form of counts and percentages. For each HV grade, we compared the mean change in SEFAS scores and EQ5D using Welch’s t-test, which accounts for unequal variances and sample sizes. A baseline imputation technique was used to compensate for missing data and a separate sensitivity analysis was also performed excluding the second subsequent HV surgery in patients who underwent bilateral HV procedures during the study period (see Tables S1 and S2 in Supplementary data) [12]. Differences between groups are reported with 95% confidence intervals (CI). A 95% confidence interval excluding differences greater than 5 SEFAS points between groups was interpreted as indicating the absence of a clinically meaningful difference. All statistical analyses were performed with R version 4.3.1 (R Foundation for Statistical Computing, Vienna, Austria).

### Ethics, registration, data sharing plan, funding, and disclosures

This study was approved by the Swedish Ethical Review Board (Etikprövningsmyndigheten) (Reference No. 2019-02733) and conducted in accordance with the Declaration of Helsinki, plus Swedish and EU data protection regulations. All patients are informed about Swefoot and can opt out at any time. Individual consent was not required for this register-based study under Swedish law. Data access beyond the study team requires approval from the Ethical Review Authority and the register. Swefoot is approved by the Swedish Data Inspection Board and operates in accordance with Swedish legislation, i.e., the Swedish Personal Data Act.

The study received support from “Stiftelsen för Skobranschens utvecklingsfond.” Swefoot is funded by the Swedish Association of Local Authorities and Regions. Funders did not influence study design, data collection, analysis, interpretation, manuscript composition, or any other study aspects. CB had a consultancy agreement with Johnson & Johnson that was not involved in this study. The remaining authors report no conflicts of interest. Complete disclosure of interest forms according to ICMJE are available on the article page, doi: 10.2340/17453674.2025.44750

## Results

In our study we included patients who were treated with a distal CO between January 2014 and May 2021 (Figure). We identified 8,456 surgeries for HV with distal CO in the Swefoot Register between January 2014 and May 2021. Of these, 2,278 had first-time surgery for HV with 1-year postoperative data, and 2,259 had complete grading of HV according to Mann and Coughlin (mean age 55 [range 15–90] years, 83% women) (Table 1).

**Figure:**
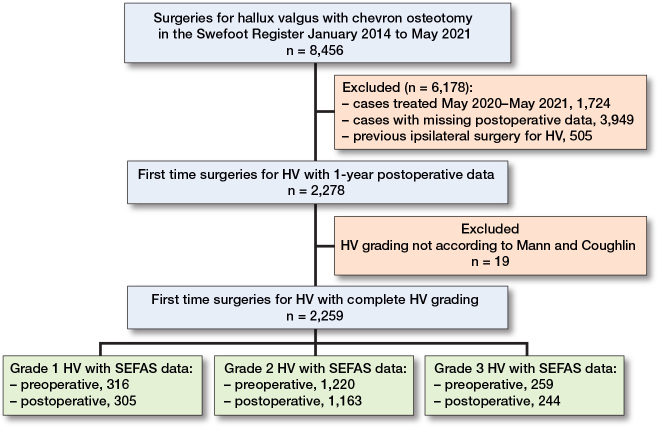
Flowchart describing the selection process for the study population (n is the number of feet).

**Table 1:** Preoperative baseline data presented as numbers (%) unless otherwise specified

Item	Grade 1	Grade 2	Grade 3
Number of feet	413	1,530	316
Number of patients	391	1,432	300
Median age (range)	52 (16–80)	55 (15–90)	63 (15–90)
Female sex	363 (88)	1,265 (83)	251 (79)
Mean body mass index (SD)	25.5 (4.2)	25.8 (4.2)	25.6 (4.2)
Diabetes mellitus	12 (2.9)	32 (2.0)	13 (4.1)
Rheumatoid arthritis	21 (5.1)	70 (4.6)	25 (8.0)
Smoker	19 (4.6)	52 (3.4)	19 (6.0)
Quit smoking before surgery	17 (4.1)	56 (3.7)	17 (5.3)

### PROMs

The mean SEFAS score increased by 11 (CI 9.8–11.8) points from the pre-surgery assessment to the 1-year post-surgery follow-up for the mild HV group, 9 (CI 9.0–10.0) for the moderate, and 9 (CI 7.5–9.9) for the severe group. The between-group differences were 1 point better (CI −2.4 to −0.2) from mild to moderate, and 2 points (CI −3.7 to −0.7) from mild to severe HV.

A sensitivity analysis excluding the second subsequent HV surgery in patients who underwent bilateral HV procedures during the study period, and who had complete SEFAS scores for 1,605 feet, revealed that SEFAS scores improved across all 3 groups (see Tables S1 and S2 in Supplementary data). Improvements in EQ-5D 3L were also observed in all 3 groups (Table 2). In addition, a baseline imputation was applied to account for missing data, and the results showed clinically relevant SEFAS and EQ-5D improvements across all HV severity grades 1 year postoperatively (see Supplementary data). Most patients reported satisfaction with the appearance of their feet after surgery.

**Table 2:** Preoperative, postoperative, and mean increase in SEFAS score and EQ-5D index from before surgery until the 1-year follow-up for the different grades of hallux valgus. PROM data is presented as means with 95% confidence interval (CI)

Factor	Grade 1	Grade 2	Grade 3	Difference Grade 2–1	Difference Grade 3–1
No. of SEFAS	305	1,163	244		
SEFAS preoperative	29 (28.4–30.1)	30 (30.0–30.9)	30 (29.5–31.4)	1 (0.2 to 2.2)	1 (–0.1 to 2.5)
SEFAS postoperative	40 (39.2–40.9)	40 (39.5–40.5)	39 (38.0–40.1)	–0.1 (–1.6 to 0.9)	–1 (–2.3 to 0.4)
Change from baseline	11 (9.8–11.8)	9 (9.0–10.0)	9 (7.5–9.8)	–1 (–2.4 to –0.2)	–2 (–3.7 to –0.7)
No of EQ-5D	284	1,119	241		
EQ5D preoperative	0.63 (0.59–0.66)	0.67 (0.65–0.70)	0.66 (0.64–0.69)	0.04 (0.01 to -0.07)	0.04 (–0.01 to 0.08)
EQ5D postoperative	0.84 (0.82–0.87)	0.85 (0.84–0.86)	0.83 (0.80–0.85)	0.01 (–0.02 to 0.03)	–0.02 (–0.05 to 0.02)
Change from baseline	0.22 (0.19–0.25)	0.18 (0.17–0.20)	0.16 (0.13–0.20)	–0.04 (–0.07 to –0.001)	–0.06 (–0.1 to –0.01)

Among patients with mild severity, 83% were satisfied, compared with 82% in those with moderate severity, and 72% in the severe group at 1-year post-surgery, demonstrating a considerable improvement from preoperative to postoperative results (Table 3). A similar trend was observed regarding satisfaction with footwear; preoperative satisfaction ranged from 26% to 37%, with Grade 3 being the least satisfied and Grade 1 the most preoperatively. However, post-surgery satisfaction with footwear improved dramatically to 83–84%, regardless of the initial grade.

**Table 3:** Patient-reported satisfaction: number and percentage satisfied or not satisfied before surgery and at the 1-year follow-up for the different grades of hallux valgus

Factor	Grade 1	Grade2	Grade 3
Satisfaction with surgery 1 year postoperatively			
No	69 (17)	279 (18)	61 (28)
Yes	344 (83)	1,247 (82)	155 (72)
Satisfaction with the appearance of foot preoperatively			
No	229 (71)	967 (78)	234 (87)
Yes	92 (29)	276 (22)	35 (13)
Satisfaction with the appearance of foot postoperatively			
No	56 (17)	250 (20)	58 (22)
Yes	265 (83)	993 (80)	211 (78)
Satisfaction with shoe wear preoperatively			
No	204 (64)	851 (69)	195 (74)
Yes	117 (37)	385 (31)	70 (26)
Satisfaction with shoe wear postoperativelly			
No	53 (17)	200 (16)	46 (17)
Yes	268 (83)	1,036 (84)	219 (83)
Forefoot pain (metatarsalgia) preoperatively			
Mild	161 (51)	592 (49)	114 (45)
Strong	152 (49)	612 (51)	139 (55)
Forefoot pain (metatarsalgia) postoperatively			
Mild	260 (83)	1,005 (83)	209 (83)
Strong	53 (17)	199 (17)	44 (17)

### HV and internal fixation

The CO was fixated with a screw or with a K-wire in 56% of all feet (n = 1,273/2,255), in 62% of the mild HV group (n = 255/412), 54% of the moderate HV group (n = 829/1,529), and 60% of the severe HV group (n = 189/314) (Table 4).

**Table 4:** Number (%) of feet reported to Swefoot with fixated or not fixated chevron osteotomy. Data is presented for all patients and separately by grades of hallux valgus

Factor	Grade 1	Grade 2	Grade 3	All grades
No. of feet	412	1,529	314	2,255
No fixation	157 (38)	700 (46)	125 (40)	982 (44)
Fixation	255 (62)	829 (54)	189 (60)	1,273 (56)

In all 3 HV grades, patients treated with fixation showed numerically greater improvement in SEFAS scores compared with those without fixation, although these differences were not statistically significant (Table 5).

**Table 5:** Comparison of SEFAS scores between hallux valgus cases with and without fixation. Values are mean SEFAS with 95% confidence interval

Grade	n	Preoperative	Postoperative	Change from baseline	Change difference ^a^
Grade 1					
Fixation	179	29 (27.7–30.0)	40 (39.2–41.5)	12 (10.3–12.8)	
No fixation	125	30 (28.5–31.2)	40 (38.2–40.1)	10 (8.3–11.2)	1.8 (–0.1 to 3.8)
Grade 2					
Fixation	576	30 (29.0–30.3)	40 (38.9–40.2)	10 (9.2–10.6)	
No fixation	577	31 (30.7–31.9)	40 (39.7–41.1)	9 (8.4–9.8)	0.8 (–0.2 to 1.8)
Grade 3					
Fixation	143	29 (28.1–30.7)	38 (37.0–39.8)	9 (7.6–10.4)	
No fixation	99	32 (30.4–33.5)	40 (38.3–41.4)	8 (6.0–9.8)	1.1 (–1.3 to 3.5)

a Between fixation and no fixation.

### Additional surgery

The distribution of Akin osteotomies and distal soft tissue procedures varied across the severity groups: 10% and 4% in the mild group, 25% and 11% in the moderate group, and 61% and 17% in the severe group, respectively (Table 6).

**Table 6:** Number (%) of additional procedures performed in the patients surgically treated with a chevron osteotomy by grade of hallux valgus

Item	Grade 1	Grade 2	Grade 3
Chevron osteotomies	413	1,530	316
Akin osteotomies	41 (10)	387 (25)	193 (61)
Soft tissue procedures	15 (3.6)	152 (11)	53 (17)

## Discussion

This study represents the largest collection of such data within a national cohort. We aimed to assess 1-year PROMs after CO for 3 severity levels, the number of additional surgical interventions during the index procedure, and the association between the presence or absence of internal fixation and PROMs. We found that the PROMs measured with SEFAS and EQ-5D 3L improved in patients who underwent distal CO for their HV, independent of the deformity’s severity. Additionally, patients who underwent CO with internal fixation showed numerically greater improvements in SEFAS scores, but not statistically significant when compared with those without internal fixation. As the severity of HV increased, there was a more frequent use of additional interventions, such as Akin osteotomies and soft tissue procedures.

A key advantage of the distal CO is its less invasive nature compared with other surgical techniques, along with minimal shortening of the metatarsal [13]. In our study, using a large, register-based cohort, we can observe patient-reported improvement after distal CO even for larger deformities.

In our study, the SEFAS summary score and the EQ-5D-3L index improved for all 3 grades of HV, exceeding the thresholds for clinical relevance [9]. Population-based age- and sex-specific normative values for the SEFAS were published by Cöster et al. in 2018 and the mean postoperative scores in the present study range between 39 and 40, i.e., close to the mean SEFAS score of the general population in the same age group [14].

From these results distal CO could be a surgical option to treat all grades of HV deformities. Schneider et al. 2004 found that the results were consistent with further improvement over a longer follow-up period in a study group of 112 feet without recurrence of the deformity [15]. We also know from other studies that the PROM scores further improve after 1 year post-surgery [16,17].

The patient-reported satisfaction was not as high as we had expected 1 year post-surgery, and in particular patients with severe deformities were less satisfied. The reasons behind these results remain unclear and require further investigation. We found that the responses regarding appearance, footwear, and forefoot pain are as important as the PROMs evaluating outcomes, and we noticed improvements from before to 1-year post-surgery. After surgical reconstruction with a distal CO, the satisfaction increased regarding the appearance of the patients’ feet but also in terms of footwear. Approximately 80% of the patients were satisfied after 1 year, but the change was greater in those with severe HV, who initially reported more dissatisfaction compared with those with mild or moderate HV. Bahar and Yildiz showed that patients’ perception of subjective pain and functional improvement were affected by visualizing improvement in the cosmetic appearance of the foot [18]. In previous publications, we did not find specific patient-reported data evaluating these important issues. Regarding forefoot pain (metatarsalgia) approximately 50% of the patients in our study had noticeable forefoot pain before surgery, but after 1 year this group had decreased to 17% in all grades of HV. Forefoot pain or metatarsalgia is discussed in previous publications, but in most of the studies iatrogenic transfer metatarsalgia is discussed and not the reduction of forefoot pain [19]. In contrast to these findings, our study demonstrated a reduction in forefoot pain following surgery. Further studies are needed to better understand the cause of metatarsalgia in HV and the possible reduction of forefoot pain post-surgery. Appearance, shoe wear, and forefoot pain are obviously important issues for this group of patients, but they are difficult to evaluate within a PROM. It may be necessary to use additional questions together with PROMs to evaluate all aspects of the disease and results post-surgery.

In all 3 HV grades, patients treated with fixation showed numerically greater improvement in SEFAS scores compared with those without fixation although the differences were not statistically significant. The necessity for fixation is controversial. Internal fixation was originally not advocated for the CO but when complications like displacements of the first metatarsal head fragment were recognized surgeons started to fixate their osteotomies more frequently [20]. Surgeons who fixate the osteotomy claim that the recurrences are reduced, the healing of the osteotomies is faster, but also that patients can be mobilized faster and that casts are not needed. The evidence for these statements is sparse. Pentikäinen et al. [21] found that the metatarsal shift was larger when the osteotomy was fixated, but they could not find differences in patient-reported outcomes after 1 year. No other randomized study has been published comparing the fixated or not fixated osteotomy. In the current study internal fixation of the CO was observed in 62% of the mild HV group, 54% of the moderate HV group, and 60% of the severe HV group. In all 3 severity grades there was an overall improvement in SEFAS scores after surgery.

A simultaneously performed Akin osteotomy, a chevron-Akin double osteotomy, has in previous studies shown improved results in comparison with an isolated CO [22]. Lechler et al. [22] found that the improvement in the HV and intermetatarsal angles, but also the reduction of the incongruent joint, was greater in patients where a double osteotomy is performed. However, no statistically significant differences could be found when analyzing patient-reported outcomes [23]. In the literature, there is no clear recommendation on when to add the Akin osteotomies. In our study, we found that these procedures were used in all degrees of HV, but the greater the deformities the more Akin osteotomies were performed. In the present study, soft tissue procedures were performed in only 3.6% of the mild cases, and in the severe grades the incidence was 17%. In previous publications the necessity of soft tissue release is pointed out and good outcomes are reported, but other studies suggest that the release may not be needed as it does not improve the correction and complications including decreased range of motion, hallux varus, and nerve damage are avoided [24-26]. In the Swedish national guidelines, soft tissue procedures are not recommended for mild or moderate HV. This study confirms that most surgeons are adhering to these recommendations.

### Strengths

The strength of this study is its national register design, meaning that data was prospectively collected in routine clinical practice. The large number of patients participating in the study enhances its statistical power, while the involvement of numerous units and surgeons mitigates the potential influence of any single operating surgeon’s role. Another strength is that we have used a foot- and ankle-specific PROM (SEFAS), which is thoroughly validated in patients with foot and ankle disorders and is a recommended PROM evaluating HV surgery [27].

### Limitations

A large amount of missing patient-reported data limits the generalizability of the results. For patients registered between May 2020 and 2021, less than a year had passed since they were operated on, which means that they had not had the chance to complete the 1-year questionnaire when the data was extracted. These patients should not be considered as dropouts because we can assume that they completed the questionnaires after we extracted data from the register. Another limitation is the incomplete coverage and completeness of the register. Swefoot is still a relatively new register, and the collection of PROMs remains challenging. As observed in other register-based studies, response rates are typically highest at baseline and decline over time. While there is no universally accepted minimum threshold for generalizability, the ISAR PROMs Working Group recommends a 60% response rate as acceptable [28]. Further limitations are that the follow-up was limited to 1 year and that we did not present adverse events in detail, revision rate, or secondary surgery because the register is too young, which means we do not have enough long-term data yet. Furthermore, inherent to our study design, no postoperative radiographic analysis to identify potential under-corrections of the HV was possible. Such under-corrections may increase the risk of recurrence and reduce patient satisfaction over a longer period. However, previous studies showing improvements in PROM scores after CO beyond 1 year post-surgery suggest that longer-term data might reveal further improvements [16]. In addition, we did not analyze the impact of Akin osteotomies or soft tissue procedures on SEFAS outcomes due to insufficient subgroup sizes for reliable statistical analysis. In future studies, using Swefoot data, we must evaluate the long-term results including recurrence of deformities, pain, and the prevalence of reoperations.

### Conclusion

Distal CO improved 1-year patient-reported outcomes across all grades of HV. Improvements were observed both with and without internal fixation. In more severe cases, additional procedures such as the Akin osteotomy and distal soft tissue release were more commonly performed.

## Supplementary Material



## Supplementary data

Supplementary Tables 1–2 are available as Supplementary data on the article page, doi: 10.2340/17453674.2025.44750

CB and MC designed the study. CB, ACS, ET, NH, and MC wrote the manuscript, read, edited, and approved the final version.

The authors would like to thank the participating patients and physicians in this register study and the Swefoot register for their cooperation in providing access to the data

Handling co-editors: Bart A Swierstra and Robin Christensen

*Acta* thanks Joris Petrus Sebastiaan Hermus and Helka Koivu for help with peer review of this manuscript.
