# Supplementary material for: Distal chevron osteotomies enhance patient-reported outcomes for all severity grades of hallux valgus: a cohort study
Source: Acta Orthop. 2025 Oct 16;96:788–94. doi: 10.2340/17453674.2025.44750 (PMC12529811; doi:10.2340/17453674.2025.44750)
Supplement: Supplementary file 1 [file ActaO-96-44750-s1.pdf]

Supplementary data

Table S1. Sensitivity analysis. Second hallux valgus surgery has been excluded in patients with bilateral hallux valgus surgery. Values are mean SEFAS with 95% confidence interval

SEFAS	Grade 1 (n = 289)	Grade 2 (n = 1,083)	Grade 3 (n = 233)
Preoperative	28 (27.5–29.3)	29 (28.9–29.8)	30 (28.6–30.6)
Postoperative	40 (39.0–40.8)	40 (39.3–40.3)	39 (37.8–39.9)
Difference	11 (10.5–12.5)	10 (9.9–11.0)	9 (8.1–10.5)

Table S2. Sensitivity analysis with baseline imputation. Values are mean score with 95% confidence interval

	Grade 1	Grade 2	Grade 3	Grade 2 – Grade 1	Grade 3 – Grade 1
No of SEFAS	316	1,220	259		
SEFAS preoperative	29 (28.3–30.0)	30 (29.9–30.7)	30 (29.6–31.4)		
SEFAS postoperative	40 (38.7–40.5)	39 (38.8–39.8)	38 (37.6–39.6)		
Change from baseline	10 (9.5–11.4)	9 (8.5–9.5)	8 (7.0–9.2)		
Change difference (CI)				–1 (–2.5 to –0.3)	–2 (–3.7 to –0.9)
No of EQ5D	318	1,215	258		
EQ5D preoperative	0.62 (0.60–0.65)	0.66 (0.65–0.68)	0.67 (0.64–0.69)		
EQ5D postoperative	0.82 (0.80–0.84)	0.83 (0.82–0.84)	0.82 (0.80–0.85)		
Change from baseline	0.20 (0.17–0.23)	0.17 (0.16–0.18)	0.15(0.12–0.19)		
Change difference				–0.03 (–0.06 to 0.01)	–0.04 (–0.09 to –0.001)
